# A predictive nomogram of thyroid nodules based on deep learning ultrasound image analysis

**DOI:** 10.3389/fendo.2025.1504412

**Published:** 2025-04-29

**Authors:** Yuan Li, Ting Li, Kai He, Xiao-xiao Cui, Lu-lu Zhang, Xiu-liang Wei, Zhi Liu, Mei Wu

**Affiliations:** ^1^ Department of Ultrasound, the Second Hospital, Cheeloo College of Medicine, Shandong University, Jinan, Shandong, China; ^2^ School of Information Science and Engineering, Shandong University, Qingdao, China; ^3^ Department of Pathology, the Second Hospital, Cheeloo College of Medicine, Shandong University, Jinan, Shandong, China

**Keywords:** thyroid nodules, ultrasound, C-TIRADS, deep learning, nomogram

## Abstract

**Objectives:**

The ultrasound characteristics of benign and malignant thyroid nodules were compared to develop a deep learning model, aiming to establish a nomogram model based on deep learning ultrasound image analysis to improve the predictive performance of thyroid nodules.

**Materials and methods:**

This retrospective study analyzed the clinical and ultrasound characteristics of 2247 thyroid nodules from March 2016 to October 2023. Among them, 1573 nodules were used for training and testing the deep learning models, and 674 nodules were used for validation, and the deep learning predicted values were obtained. These 674 nodules were randomly divided into a training set and a validation set in a 7:3 ratio to construct a nomogram model.

**Results:**

The accuracy of the deep learning model in 674 thyroid nodules was 0.886, with a precision of 0.900, a recall rate of 0.889, and an F1-score of 0.895. The binary logistic analysis of the training set revealed that age, echogenic foci, and deep learning predicted values were statistically significant (*P*<0.05). These three indicators were used to construct the nomogram model, showing higher accuracy compared to the China thyroid imaging reports and data systems (C-TIRADS) classification and deep learning models. Moreover, the nomogram model exhibited high calibration and clinical benefits.

**Conclusion:**

Age, deep learning predicted values, and echogenic foci can be used as independent predictive factors to distinguish between benign and malignant thyroid nodules. The nomogram integrates deep learning and patient clinical ultrasound characteristics, yielding higher accuracy than the application of C-TIRADS or deep learning models alone.

## 1 Introduction

Thyroid nodules are common clinical findings and have a prevalence rate ranging from 34-66%, showing regional differences (1). In China, the prevalence of thyroid nodules is nearly 40%, with women exhibiting significantly higher rates than men (2). The incidence rate of thyroid cancer is about 7-15%. The most common pathological type is papillary thyroid cancer, accounting for 80-90% of all thyroid malignant tumors (3, 4). Since the 1980s, the incidence rate of thyroid cancer has gradually increased, but the mortality rate has remained relatively stable and the prognosis is usually good. Therefore, excessive diagnosis and treatment should be avoided (4, 5). At present, ultrasound remains the best imaging method for the thyroid gland and plays an essential role in the diagnosis of thyroid nodules (3). To standardize thyroid ultrasound results, various thyroid imaging reports and data systems (TI-RADS) have been proposed, such as the American Society of Radiology TI-RADS (ACR TI-RADS) (6), the European Thyroid Association TI-RADS (EU-TIRADS) (7), and the Korean Society of Thyroid Radiology TI-RADS (K-TIRADS) (8). In 2020, the Chinese Medical Association proposed the Chinese guidelines, also known as C-TIRADS, for ultrasound malignancy risk stratification of thyroid nodules, which were developed based on China’s national conditions and medical status (9). Traditionally, ultrasound examination has been shown to be highly subjective, depending on the operator’s ability. Different physicians may report different descriptions of the same ultrasound features (10, 11). However, fine needle aspiration for pathological examination of thyroid nodules is the gold standard for the diagnosis of thyroid nodules (12).

In recent years, due to the improvement in computing power and the availability of large-scale data, artificial intelligence, represented by deep learning has emerged as an essential tool in the field of medical imaging. Deep learning algorithms have promoted the development of precision medicine (13, 14). At present, deep learning has been widely applied in the ultrasound diagnosis of thyroid diseases, improving the classification, segmentation, and detection of thyroid images. These advances have facilitated the differential diagnosis of thyroid nodules (15, 16), the prediction of cervical lymph node metastasis (17, 18) or distant metastasis of thyroid cancer (19), and the analysis of the prognosis of thyroid cancer (20). Traditional convolutional neural networks (CNNs) represent an integral component of medical image analysis (21–25). However, due to the internal limitations of the algorithm, CNNs cannot model long-range dependencies. CNNs only focus on local pixels in the entire image, analyzing local features rather than learning global patterns (26). The Vision Transformer (ViT) model is a deep neural network based on an attention model proposed by Alexey Dosovitskiy. Its main feature is its ability to effectively store global structural information of images, which has been proven to be superior to the CNN models in the field of medical imaging (27, 28). This study uses ViT algorithm and aims to establish a nomogram based on deep learning ultrasound image analysis, integrating clinical data, ultrasound features, and deep learning results of thyroid nodules to assist in predicting the diagnosis of thyroid nodules.

## 2 Materials and methods

### 2.1 General information and grouping of patients

This retrospective study included 2247 cases of thyroid nodules (including 927 benign nodules and 1320 malignant nodules) treated at the Second Hospital of Shandong University from March 2016 to October 2023. All patients underwent thyroid ultrasound examinations before surgery or puncture. The digital ultrasound images were gathered from the ultrasound workstation.

Inclusion criteria (1): Preoperative or pre-puncture thyroid ultrasound examination; (2) Clear ultrasound image, with complete transverse and longitudinal images of the same nodule; (3) Complete clinical data; (4) Clear pathological diagnosis after surgery or puncture. Exclusion criteria: (1) Multiple (more than one) nodules on the same ultrasound section; (2) The required image section overlaps with measurement scales, Color Doppler Flow Imaging (CDFI) information, or elastography information, etc.; (3) Nodule puncture was performed before the ultrasound examination in our hospital; (4) Incomplete clinical data; (5) The pathological diagnosis is unclear.

**Figure 1:**
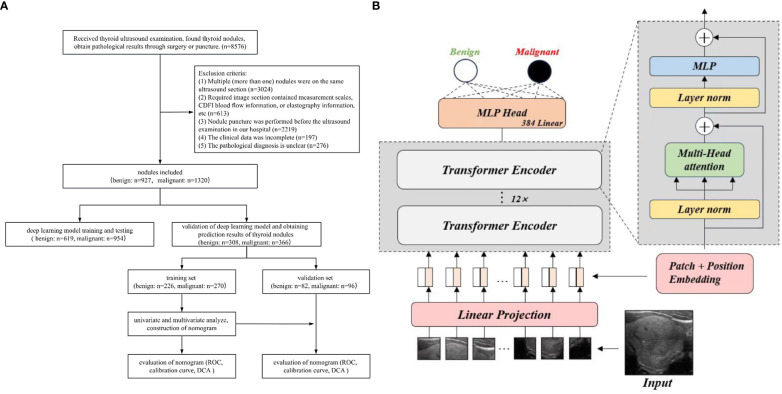
Flowchart. **(A)** Flowchart of the study. **(B)** Flowchart of ViT model.

All nodules were randomly divided in a 7:3 ratio, with 1573 nodules used for training and testing the deep learning model and 674 nodules used for validation. A external public database TN3K (29) was also used for testing; then the deep learning prediction results were obtained for the benign and malignant nodules. Thereafter, 674 nodules were divided into the benign group (308 cases) and the malignant group (366 cases) based on pathological results. About 70% of benign and malignant nodules were randomly selected as the training set to construct a nomogram chart, and 30% of benign and malignant nodules were assigned to the validation set to evaluate the nomogram chart. This study was approved by the Ethics Review Committee of the Second Hospital of Shandong University (KYLL2024752), and all patients provided signed informed consent. All procedures were conducted in compliance with the Helsinki Declaration. The flowchart of this study is shown in **Figure 1A**.

### 2.2 Analysis of ultrasound images

The ultrasound diagnostic instruments included GE Logic E9 (linear probe, frequency 9-15MHz, Wauwatosa, America) and Mindray Resona 7S (linear probe, frequency 9-14MHz, Shenzhen, China). The patient was placed in the supine position with excessive neck extension, fully exposing the anterior cervical area, and a comprehensive scan of the thyroid gland was performed. Two physicians with over five years of experience in ultrasound diagnosis conducted a retrospective analysis of ultrasound images of thyroid nodules based on the C-TIRADS criteria (9), recording the size (maximum diameter of the nodule), location (upper lobe, middle lobe, lower lobe, and isthmus), orientation (vertical, horizontal), margin (clear, unclear), shape (regular, irregular), internal composition (solid, solid-cystic, cystic, spongiform), echogenicity (anechoic, hyperechoic, isoechoic, hypoechoic, markedly hypoechoic), echotexture (homogeneous, heterogeneous), echogenic foci (no echogenic foci or comet-tail artifacts, macrocalcifications and peripheral calcifications, microcalcifications and punctate echogenic foci), halo (absent halo, even thickness halo, uneven thickness halo), posterior feature (no posterior feature, enhancement, shadowing), and relationship with the capsule (distant, adjacent, or breakthrough). Moreover, the C-TIRADS classification of thyroid nodules was determined. Among them, nodules classified as class 3 or below by C-TIRADS were defined as benign; in contrast, nodules with a C-TIRADS classification of class 4a or above indicate ultrasound malignancy diagnosis. During this process, both physicians were blinded to the pathological results. Discrepancies in the ultrasound classification between the two physicians were settled by discussion until a consensus was reached to determine the final category of the nodule. While reviewing the images, the two doctors selected transverse and longitudinal images of each nodule to prepare for the training of the deep learning model.

### 2.3 Deep learning algorithms

In this study, we employed a transformer-based approach to classify thyroid nodules into two categories. Thyroid ultrasound images were first cropped to a uniform 224×224 pixel size and then processed using the ViT model (27), which divided each image into 16×16 pixel patches. These patches were flattened from the original (H×W×C) format into a sequence with shape N×(P^2^×C) (where N = HW/P^2^) and projected into D dimensions via a trainable linear layer, with a learnable embedding prepended to preserve spatial information. Thyroid ultrasound images were subsequently processed through Transformer blocks that leverage multi-head self-attention, layer normalization, and residual connections for deep feature extraction and contextual modeling. Finally, the combined outputs were passed through a two-layer MLP with GELU activation to generate global representations for effective binary classification.

To train the model, we used the Adam optimizer. This optimizer was configured with a momentum of 0.9 to ensure stable learning and a weight decay of 0.05 to help prevent overfitting. Additionally, we applied a dynamic learning rate strategy—starting at 0.0004 and gradually decreasing it following a cosine schedule—to facilitate a smoother training process. The training was conducted over 150 epochs with a batch size of 24 images per iteration.

All experiments were implemented using the PyTorch 2.1.1 and Timm 0.9.1 frameworks on an Ubuntu 18.04 system. The computational setup included an Intel Xeon Gold 6230 CPU running at 2.10 GHz and an NVIDIA GeForce RTX 3090 GPU with 24GB of memory, ensuring robust performance for our deep learning tasks. The flowchart of deep learning model is shown in **Figure 1B**.

### 2.4 Statistical methods

SPSS 21.0 statistical software was used for analysis. Categorical variables were presented as numbers and percentages and analyzed by the χ^2^ test and Fisher’s exact test. Continuous variables were analyzed using a single sample K-S test to determine if the variables followed a normal distribution. Variables conforming to a normal distribution were analyzed using a t-test. In contrast, variables not conforming to a normal distribution were analyzed using the U-test. Multivariate analysis was conducted using logistic regression analysis. R (4.2.3) was used to construct and evaluate nomogram model. The receiver operating characteristic (ROC) curves of the model were plotted, and the area under the curve (AUC) and 95% confidence interval (CI) were calculated to evaluate the predictive ability of the models. Furthermore, the DeLong test was used to determine the statistical significance of differences in AUC between different models. A calibration curve was constructed to evaluate the calibration degree of the model and decision curve analysis (DCA) was performed to evaluate clinical benefits. The online interactive nomogram was constructed by Shinny. Violin plots were used to illustrate the age distribution differences between patients with benign and malignant nodules. *P*<0.05 indicated statistical significance.

## 3 Results

### 3.1 General features of deep learning model training and validation sets

Statistically significant differences (*P*<0.05) in age, size, and C-TIRADS classification were observed between benign and malignant nodules in both the deep learning model training set and the validation set. The general features of the deep learning model training and validation sets are shown in **Table 1**. The exact pathological types of all thyroid nodules are shown in **Supplementary Table 1**.

**Table 1:** General features of deep learning model training and validation sets.

	Deep learning model training set (n=1573)		*T/U/χ^2^*	*P*	Deep learning model validation set (n=674)		*T/U/χ^2^*	*P*
	Benign (n=619)	Malignant (n=954)			Benign (n=308)	Malignant (n=366)		
Sex			2.462	0.117			0.942	0.332
Male	119 (19.2%)	215 (22.5%)			62 (20.1%)	63 (17.2%)		
Female	500 (80.8%)	739 (77.5%)			246 (79.9%)	303 (82.8%)		
Age	52.80 ± 11.89	46.79 ± 11.45	10.017	<0.001^*^	51.30 ± 12.66	45.86 ± 12.36	5.631	<0.001^*^
Size(cm)	1.3 (0.7, 2.9)	0.7 (0.5, 1.2)	11.542	<0.001^*^	1.0 (0.6, 2.0)	0.7 (0.5, 1.0)	5.726	<0.001^*^
C-TIRADS			472.573	<0.001^*^			156.155	<0.001^*^
Benign	322 (52.0%)	44 (4.6%)			137 (44.5%)	15 (4.1%)		
Malignant	297 (48.0%)	910 (95.4%)			171 (55.5%)	352 (95.9%)		

*C-TIRADS* China thyroid imaging reports and data systems.

^*^ *P*<0.05.

### 3.2 Prediction performance of the deep learning model

**Figure 2:**
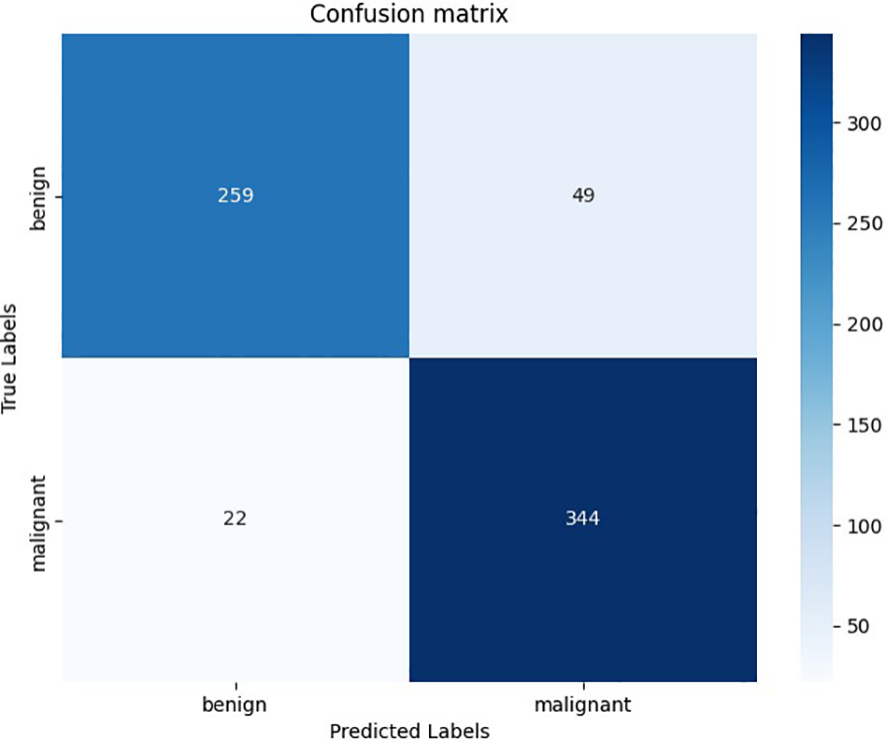
The confusion matrix of deep learning models in the validation set. The horizontal axis represents the prediction results of the deep learning model, and the vertical axis represents the pathological results.

The accuracy of this model in the validation set of 674 nodules is 0.886, while the accuracy of TN3K is 0.825. This model showed high precision, recall rate, and F1-score in all nodules, benign nodules, and malignant nodules in the validation set respectively (**Table 2**). The confusion matrix of 674 nodules in the model is shown in **Figure 2**.

**Table 2:** Prediction performance of deep learning models in validation sets.

	precision	recall	F1-score
All nodules	0.900	0.889	0.895
Benign nodules	0.870	0.880	0.880
Malignant nodules	0.900	0.890	0.890
TN3K	0.770	0.687	0.726

### 3.3 Clinical-ultrasound features of thyroid nodules identified from the nomogram training set and validation set

In the nomogram training set, 14 indicators, including age, size, orientation, location, internal composition, echogenicity, shape, margin, echotexture, posterior features, echogenic foci, halo, relationship with the capsule, and deep learning predicted values, showed statistically significant differences (*P*<0.05) between the benign and malignant groups, while gender showed no statistically significant difference. In the nomogram validation set, 13 indicators, including age, orientation, location, internal composition, echogenicity, shape, margin, echotexture, posterior features, echogenic foci, halo, relationship with the capsule, and deep learning predicted values showed statistically significant differences (*P*<0.05) between the benign and malignant groups. However, no statistically significant difference was observed in patient gender and size. The clinical-ultrasound characteristics analysis of thyroid nodules in the nomogram training set and validation set are shown in **Table 3**.

**Table 3:** The clinical-ultrasound characteristics analysis of thyroid nodules in the nomogram training set and validation set.

	Nomogram training set (n=496)		*T/U/χ^2^*	*P*	Nomogram validation set (n=178)		*T/U/χ^2^*	*P*
	Benign (n=226)	Malignant (n=270)			Benign (n=82)	Malignant (n=96)		
Sex			0.872	0.350			0.110	0.740
Male	45 (19.9%)	45 (18.1%)			17 (20.7%)	18 (18.8%)		
Female	181 (80.1%)	225 (81.9%)			65 (79.3%)	78 (81.2%)		
Age	51.00 ± 12.74	45.14 ± 12.44	5.163	<0.001*	52.13 ± 12.46	47.88 ± 11.96	2.323	0.021*
Size (cm)	1.10 (0.60, 2.10)	0.70 (0.50, 1.10)	5.619	<0.001*	0.80 (0.50, 1.65)	0.70 (0.50, 0.98)	1.708	0.088
Orientation			65.495	<0.001*			8.531	0.003*
Horizontal	181 (80.1%)	122 (45.2%)			53 (64.6%)	41 (42.7%)		
Vertical	45 (19.9%)	148 (54.8%)			29 (35.4%)	55 (57.3%)		
Location			18.401	<0.001*			8.270	0.041*
Upper lobe	22 (9.7%)	59 (21.9%)			11 (13.4%)	19 (19.8%)		
Middle lobe	134 (59.3%)	128 (47.4%)			51 (62.2%)	48 (50.0%)		
Lower lobe	60 (26.5%)	60 (22.2%)			20 (24.4%)	22 (22.9%)		
Isthmus	10 (4.5%)	23 (8.5%)			0 (0.0%)	7 (7.3%)		
Internal composition			99.120	<0.001*			26.069	<0.001*
Cystic	19 (8.4%)	0 (0%)			6 (7.3%)	0 (0%)		
Spongiform	13 (5.8%)	1 (0.4%)			2 (2.5%)	0 (0%)		
Solid-cystic	47 (20.8%)	3 (1.1%)			10 (12.2%)	1 (1.0%)		
Solid	147 (65.0%)	266 (98.5%)			64 (78.0%)	95 (99.0%)		
Echogenicity			153.973	<0.001*			41.851	<0.001*
Anechoic	19 (8.4%)	0 (0%)			6 (7.3%)	0 (0%)		
Hyperechoic/isoechoic	65 (28.8%)	5 (1.9%)			19 (23.2%)	1 (1.0%)		
Hypoechoic	135 (59.7%)	162 (60.0%)			51 (62.2%)	61 (63.5%)		
Markedly hypoechoic	7 (3.1%)	103 (38.1%)			6 (7.3%)	34 (35.4%)		
Shape			162.150	<0.001*			62.862	<0.001*
Regular	151 (66.8%)	31 (11.5%)			52 (63.4%)	7 (7.3%)		
Irregular	75 (33.2%)	239 (88.5%)			30 (36.6%)	89 (92.7%)		
Margin			130.516	<0.001*			38.267	<0.001*
Clear	144 (63.7%)	38 (14.1%)			46 (56.1%)	12 (12.5%)		
Unclear	82 (36.3%)	232 (85.9%)			36 (43.9%)	84 (87.5%)		
Echotexture			89.009	<0.001*			32.320	<0.001*
Homogeneous	71 (31.4%)	3 (1.1%)			26 (31.7%)	1 (1.0%)		
Heterogeneous	155 (68.6%)	267 (98.9%)			56 (68.3%)	95 (99.0%)		
Posterior feature			30.204	<0.001*			11.969	0.003*
No	99 (43.8%)	130 (48.1%)			38 (46.3%)	47 (49.0%)		
Enhancement	77 (34.1%)	39 (14.4%)			23 (28.0%)	9 (9.4%)		
Shadowing	50 (22.1%)	101 (37.5%)			21 (25.7%)	40 (41.6%)		
Echogenic foci			85.570	<0.001*			23.604	<0.001*
No echogenic foci or comet-tail artifacts	160 (70.8%)	96 (35.6%)			56 (68.3%)	37 (38.5%)		
Macrocalcifications and peripheral calcifications	33 (14.6%)	27 (10.0%)			15 (18.3%)	14 (14.6%)		
Microcalcifications and punctate echogenic foci	33 (14.6%)	147 (54.4%)			11 (17.4%)	45 (46.9%)		
Halo			26.307	<0.001*			9.515	0.009*
Absent	187 (82.7%)	256 (94.8%)			67 (81.7%)	89 (92.7%)		
Uneven thickness	18 (8.0%)	13 (4.8%)			5 (6.1%)	6 (6.3%)		
Even thickness	21 (9.3%)	1 (0.4%)			10 (12.2%)	1 (1.0%)		
Relationship with capsule			62.638	<0.001*			23.716	<0.001*
Distant	168 (74.3%)	120 (44.4%)			63 (76.8%)	43 (44.8%)		
Adjacent	58 (25.7%)	106 (39.3%)			19 (23.2%)	39 (40.6%)		
Breakthrough	0 (0%)	44 (16.3%)			0 (0.0%)	14 (14.6%)		
Deep learning predicted value			323.124	<0.001*			97.393	<0.001*
Benign	190 (84.1%)	12 (4.4%)			69 (84.1%)	10 (10.4%)		
Malignant	36 (15.9%)	258 (95.6%)			13 (15.9%)	86 (89.6%)		

*P<0.05. Bold values: P<0.05.

### 3.4 Binary logistics regression analysis and establishment of the nomogram model

**Figure 3:**
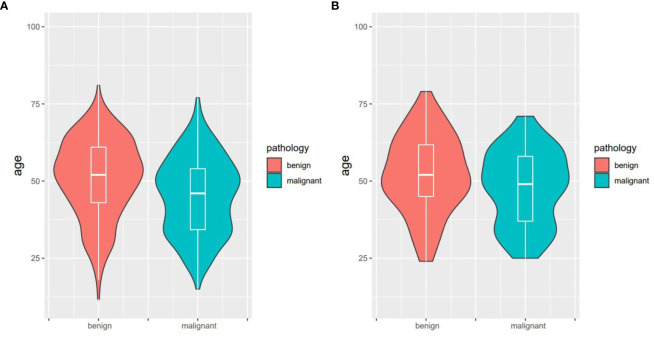
Violin plot of the age distribution of benign and malignant nodules. **(A)** in the training set; **(B)** in the validation set. The white solid line and box in the figure represent the quartiles of age.

The significant indicators in the single factor analysis of the training set were included in the binary logistics regression analysis model, showing statistical significance (χ^2^ = 499.321, *P*<0.001). The independent variables included in the model, age, deep learning predicted values, and echogenic foci (*P*<0.05). Specifically, the deep learning malignant predicted value, microcalcifications or punctate echogenic foci within nodules were indicative of malignant nodules. Moreover, every 1-year-old increase in age resulted in a 0.04 times reduction in the risk of malignant nodules. The age distribution of benign and malignant nodules, as depicted in the violin plots, followed a normal distribution with similar trends. Patients with malignant nodules were younger than those with benign nodules in both the training sets (**Figure 3A**) and validation sets (**Figure 3B**). The results of binary logistics regression analysis on the training set are shown in **Table 4**.

**Table 4:** Binary logistics regression analysis results of training set.

	β	*P*	OR	95% *CI* of OR	
				Lower limit	Upper limit
age	-0.038	0.023^*^	0.963	0.931	0.995
Deep learning malignant prediction value	4.658	0.000^*^	105.440	35.935	309.375
microcalcifications or punctate echogenic foci	1.816	0.000^*^	6.149	2.230	16.951

*OR*, Odds ratio; *CI*, confidence interval. *P<0.05.

**Figure 4:**
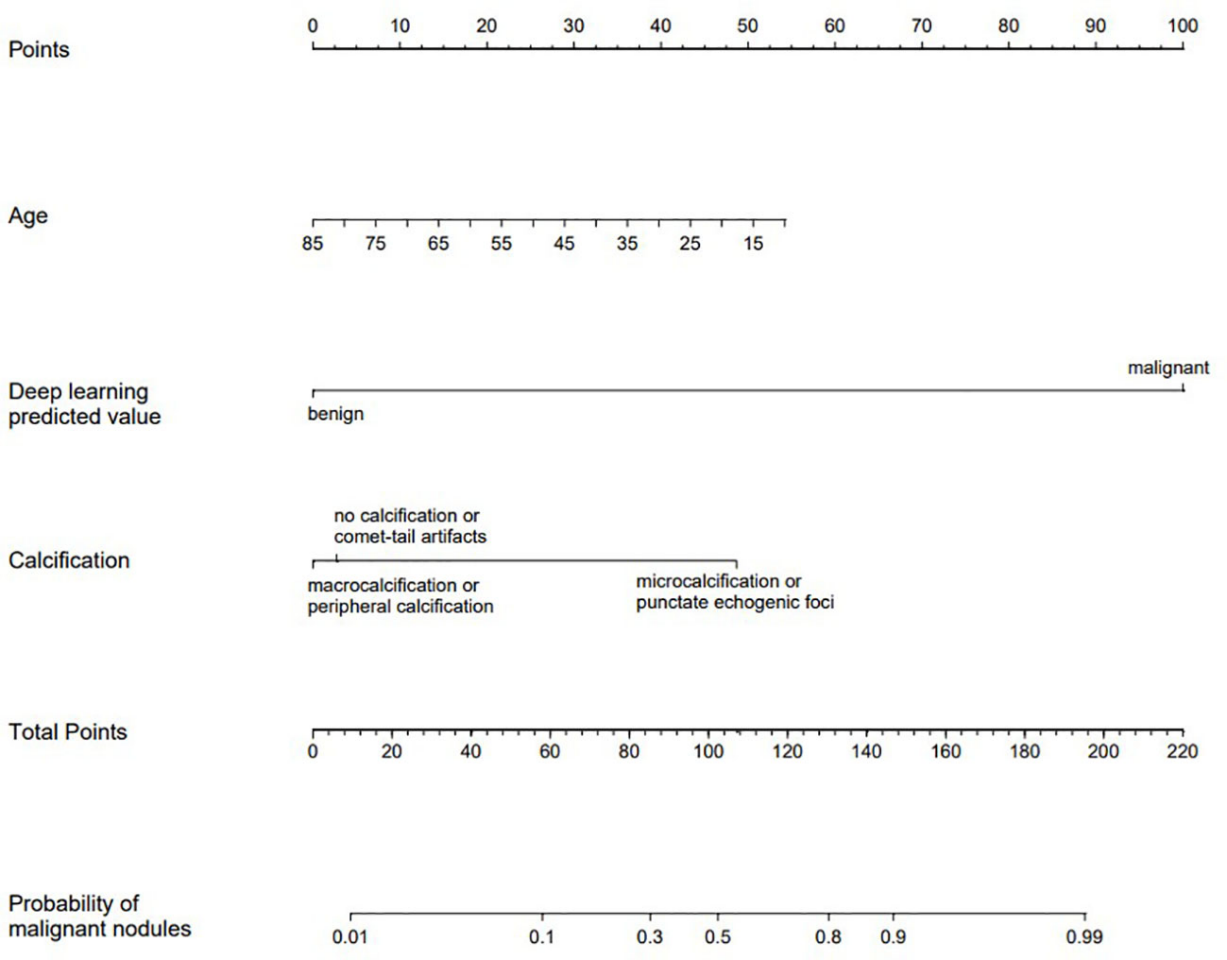
Nomogram model.

**Figure 5:**
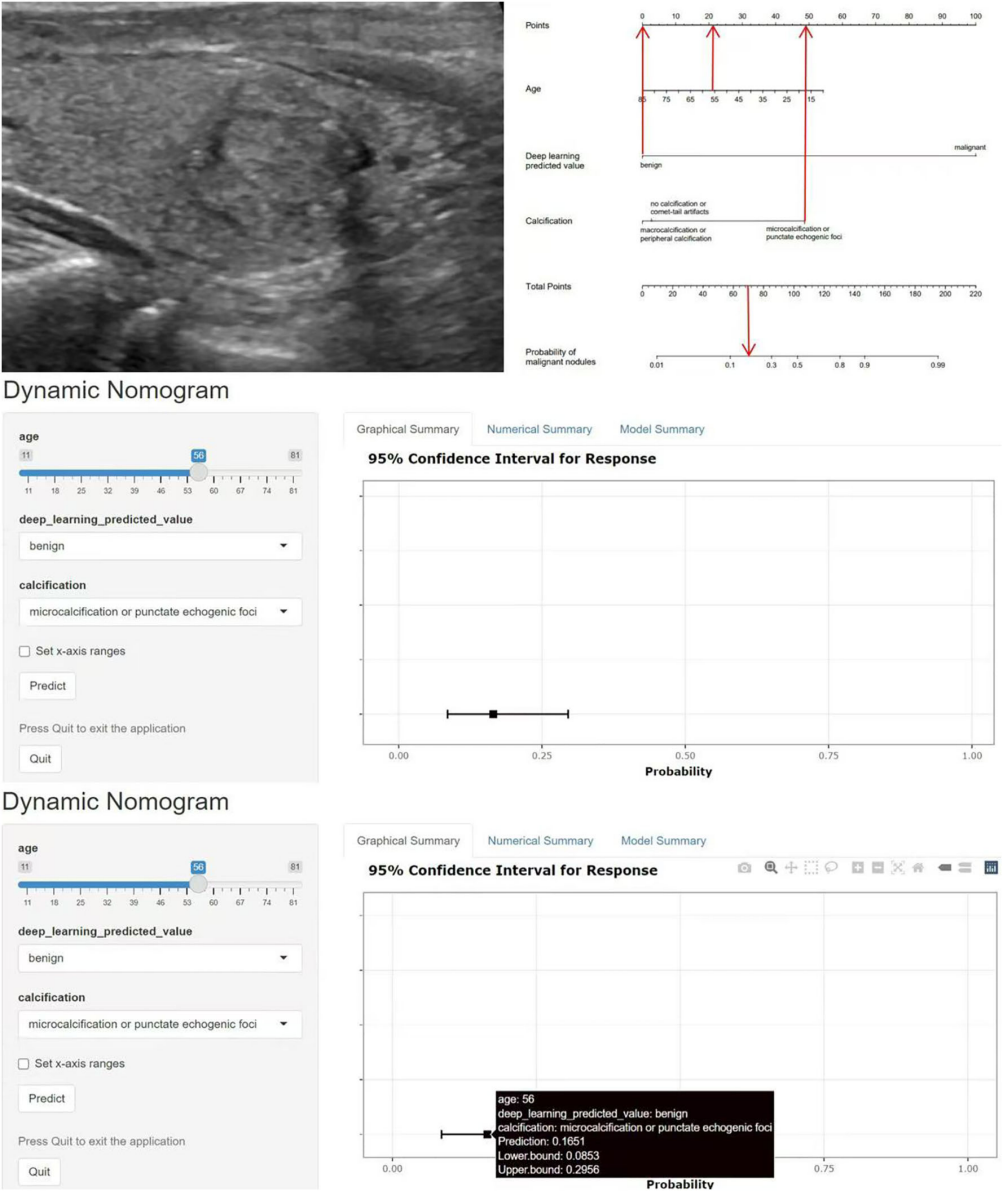
Example of application of the nomogram model. A 56-year-old female with a solid nodule in the middle of the right lobe of the thyroid gland, measuring 1.7x1.5cm. The nodule was vertically oriented, with unclear margins and microcalcifications. The C-TIRADS classification was 4c, and the deep learning model predicted a benign nodule. The total score of the nomogram model was about 70 points, corresponding to a malignant prediction probability of 0.17, and the prediction result was benign. Pathological result: nodular goiter. *C-TIRADS* China thyroid imaging reports and data systems.

These indicators were incorporated into the nomogram model, and the malignant probability of nodules was predicted. The nomogram model was shown in **Figure 4**. Furthermore, an online interactive nomogram was developed(https://saprediction.shinyapps.io/DynNomapp/). The score of individual thyroid nodules can be obtained through each indicator, with the total score of each indicator corresponding to the probability of the nodule being diagnosed as malignant (**Figure 5**).

### 3.5 Evaluation of the nomogram model

**Figure 6:**
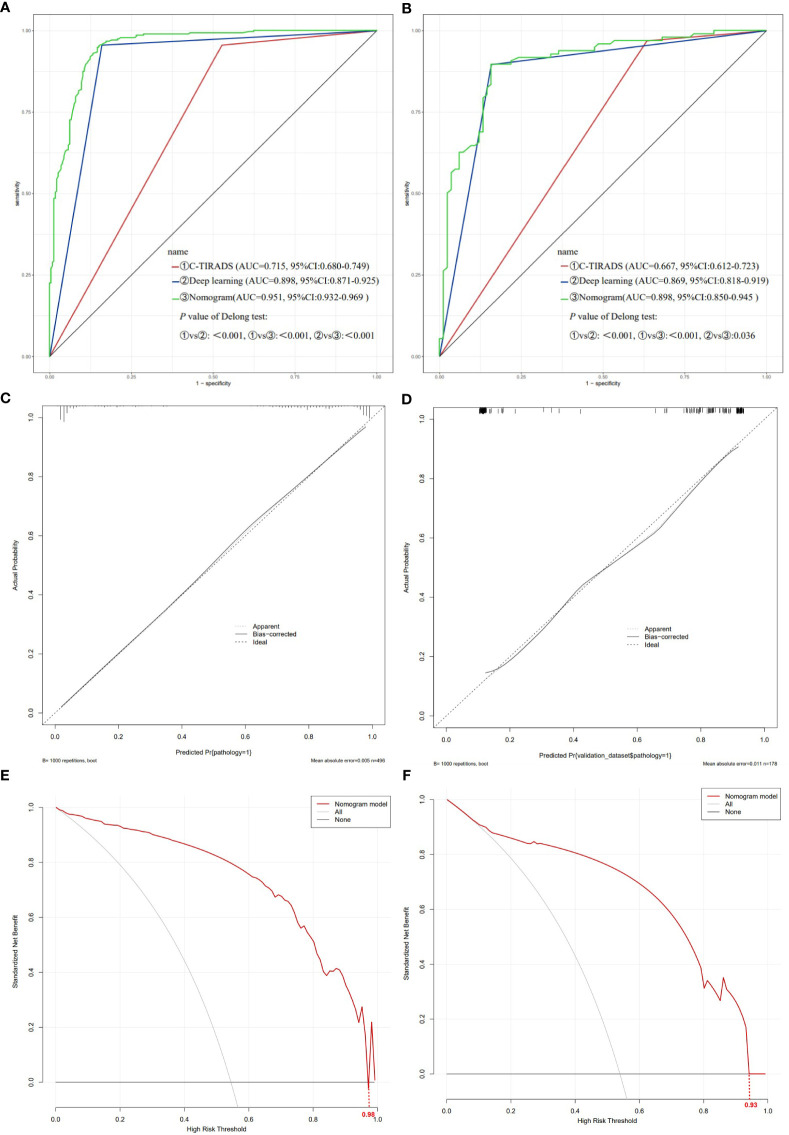
Evaluation of the nomogram model. **(A)** ROC curve of training set; **(B)** ROC curve of validation set; **(C)** Nomogram model calibration curve of training set; **(D)** Nomogram model calibration curve of validation set; **(E)** Nomogram model DCA of training set; **(F)** Nomogram model DCA of validation set. *ROC* receiver operating characteristic, *DCA* decision curve analysis.

The ROC curves of the training and validation sets revealed that the model has good accuracy. The AUC of the training set (**Figure 6A**): C-TIRADS 0.715 (95% CI: 0.680-0.749), deep learning 0.898 (95% CI: 0.871-0.925), nomogram model 0.951 (95% CI: 0.932-0.969). Validation set AUC (**Figure 6B**): C-TIRADS 0.667 (95% CI: 0.612-0.723), deep learning 0.869 (95% CI: 0.818-0.919), nomogram model 0.898 (95% CI: 0.850-0.945). The Delong test showed statistical differences (*P*<0.05) between the ROC curves. The calibration curves of the training set (**Figure 6C**) and validation set (**Figure 6D**) showed that the model has good calibration accuracy, and DCA indicated that the model has clinical benefits in both the training set (**Figure 6E**, threshold 0-0.98) and validation set (**Figure 6F**, threshold 0-0.93).

## 4 Discussion

In this study, a deep learning model was trained to comprehensively analyze the clinical and ultrasound characteristics of thyroid nodules. Based on the prediction results of the deep learning model for thyroid nodules, a nomogram model was developed and validated, which includes an online interactive nomogram, to predict the risk of malignancy of thyroid nodules. The nomogram showed good accuracy, calibration, and clinical value.

In this study, age was identified as a predictive factor for determining the benign or malignant nature of thyroid nodules. The violin plot of age distribution revealed that patients with malignant nodules were younger compared to those with benign nodules. However, the role of age in the differentiation of benign and malignant thyroid nodules remains controversial. In some previous studies, age was identified as an independent predictor of malignancy (30, 31), whereas other studies reported that age does not have statistical significance in the predictive models. This discrepancy may be attributed to the different attitudes of young and elderly patients toward the treatment of C-TIRADS 4a-5 nodules. Young patients may prefer surgical treatment, while elderly patients may prefer conservative treatment, resulting in missing pathological results (32). Therefore, the differential value of age in distinguishing benign and malignant thyroid nodules varies in different studies.

Ultrasound examination, as a non-invasive imaging modality, remains the most widely used initial examination method for thyroid assessment (3, 33). In our study, malignant and benign thyroid nodules showed statistical differences in nodule size, location, orientation, internal composition, echogenicity, shape, margin, echotexture, posterior feature, echogenic foci, halo, and capsule relationship, which is consistent with C-TIRADS (9). Among them, microcalcifications or punctate echogenic foci were found to be independent risk factors for malignant nodules. However, some benign nodules in this study also exhibited vertical growth, markedly hypoechoic features, irregular shape, unclear margin, microcalcifications, and posterior shadow, which may lead to higher C-TIRADS classification in ultrasound diagnosis, resulting in higher sensitivity and lower specificity of the C-TIRADS classification. Moreover, ultrasound examination involves a certain degree of subjectivity and relies heavily on the diagnostic experience of the physician (33). Therefore, more objective tools are required to eliminate potential observer bias, and assist in the ultrasound diagnosis of thyroid nodules. Deep learning constructs models by automatically learning features from data layers, which can automatically extract deep features from images without the need for manual intervention, reducing the subjective influence of doctors (34).

Nomogram is a commonly used visualization tool in medical research, which integrates different variables to generate the probability of clinical events, with accuracy and intuitiveness (35, 36). Previous studies have shown that a nomogram model that integrates clinical, ultrasound, and deep learning models is superior to ultrasound features or deep learning alone in identifying the nature of thyroid nodules (31, 34, 37). Du et al. (34) analyzed ultrasound images of 1076 cases of thyroid nodules and constructed a nomogram model based on deep learning, which showed high diagnostic performance (AUC>0.9). Zhang et al. (31) collected ultrasound images of 500 thyroid nodules in a similar retrospective study and performed deep learning of thyroid ultrasound images with the YOLOv3 model, and constructed a nomogram model to improve prediction ability. The model achieved 84% accuracy in identifying TI-RADS category 4 thyroid nodules. Zhong et al. (37) constructed a clinical-ultrasound-radiomics nomogram to differentiate between benign and malignant indeterminate cytology thyroid nodules, with higher accuracy than a single clinical or radiomics model. Our study incorporated clinical information of thyroid nodule patients, ultrasound features of nodules, and deep learning prediction results into a nomogram model. The ROC curve of the model reached an AUC of 0.898 in the validation set. The predictive ability of the model was improved compared to C-TIRADS and the application of deep learning models alone, which was consistent with previous research results. But compared with these, our study included a larger total number of ultrasound images and a larger sample size of ultrasound images was used for deep model training. Moreover, the present study developed an online interactive nomogram, directly displaying the malignancy probability of nodules, eliminating the step of adding scores in traditional nomograms. It can be used as a more convenient tool in clinical practice.

The diagnosis of thyroid diseases is facilitated by the comprehensive analysis of clinical, morphological, molecular, and epigenetic features using artificial intelligence algorithms (12). Therefore, the combination of deep learning models and clinical ultrasound features in this study is of great significance. Future studies can improve the predictive model of this study by incorporating pathological indicators and optimizing the model. Nevertheless, the limitations of the present study should be acknowledged. Firstly, as a single-center retrospective study, this study has certain biases and lacks validation with large sample data from multiple centers. This requires further improvement of multi-center data in our future research work. Secondly, this study only included two-dimensional grayscale ultrasound information and lacked multimodal ultrasound images and dynamic images, which will be further optimized in our future research.

## 5 Conclusion

Age, deep learning predicted values, and echogenic foci can be used as independent predictive factors for the benign or malignant judgment of thyroid nodules. The deep learning models showed superior diagnostic accuracy compared to the C-TIRADS classification. The nomogram integrates deep learning and clinical-ultrasound characteristics, yielding a higher accuracy than C-TIRADS or deep learning models alone. The online interactive nomogram provides a more convenient tool for clinical practice.

## Data Availability

The raw data supporting the conclusions of this article will be made available by the authors, without undue reservation.
