# Supplementary material for: A predictive nomogram of thyroid nodules based on deep learning ultrasound image analysis
Source: Front Endocrinol (Lausanne). 2025 Apr 29;16:1504412. doi: 10.3389/fendo.2025.1504412 (PMC12069047; doi:10.3389/fendo.2025.1504412)
Supplement: Supplementary file 1 [file DataSheet1.pdf]

Supplementary table 1 Pathological types of thyroid nodules

Pathological types	Total (n=2247)	Deep learning model training set (n=1573)	Deep learning model validation set (n=674)
Papillary thyroid carcinoma	1308	940 (59.8%)	368 (54.6%)
Thyroid follicular carcinoma	7	7 (0.4%)	0 (0)
medullary thyroid carcinoma	6	6 (0.3%)	0 (0)
Follicular adenoma	69	55 (3.5%)	14 (2.1%)
nodular goiter	751	521 (33.1%)	230 (34.1%)
Hashimoto's thyroiditis	89	30 (1.9%)	59 (8.8%)
subacute thyroiditis	7	5 (0.3%)	2 (0.3%)
Other benign conditions	10	9 (0.7%)	1 (0.1%)
